# The Challenge of Exposing and Ending Health Inequalities through Social and Policy Change: Canadian Experiences

**DOI:** 10.1177/27551938221148376

**Published:** 2023-01-05

**Authors:** Arnel M. Borras

**Affiliations:** 1 Rankin School of Nursing, St. Francis Xavier University, Antigonish, Nova Scotia, Canada

**Keywords:** health inequities, social determinants of health, critical political economy, neoliberal capitalism, colonialism, racism, sexism, public policy, labor union, social movement

## Abstract

The Canadian health system is often perceived as excellent. However, a closer examination of the political economy of health in Canada shows a radically different picture. It is a picture of persistent inequality and a history of the inability to address such inequality. Despite numerous public policy interventions to address preventable health inequalities—that is, health inequities—this societal problem persists. This research addresses how and why health inequities, especially class, race/ethnicity, and gender health inequities, persist in Canada and how to reduce such differences through public policy action. To address these questions, I performed a critical realist review, focusing on the political economy of health and policy change. Then I conducted a thematic analysis of the interview data gathered from 23 semi-structured interviews with leading Canadian policy academics, activists, and advocates. The results demonstrate that the capitalist economic system; the co-constitutives of capitalism, namely colonialism, racism, and sexism; and maldistributive public policies primarily cause health inequities in Canada. Canada's health inequities reduction requires pushing for redistributive public policies; uniting and strengthening labor unions, civil society groups, and social movements; and engaging in electoral politics. Reducing health inequities may involve struggling within and against capitalism and struggling for socialism.

## Introduction

In popular media and academic literature, the Canadian health system is often portrayed as excellent and superior, the object of envy of many countries and societies. However, a closer examination of the political economy of Canadian health shows a radically different picture. It is a picture of persistent inequality and a history of the inability to address such inequality. These realities have been further exposed during the pandemic. How and why is this so, and how can the historical inequality in health and health care be finally broken? This article aims to address some of these key questions.

Classic and recent health studies literature provide empirical evidence on health inequalities among and within countries.^1–3^ Studies have considered the causes and barriers to addressing health inequalities and suggested various ways to address them.^4–7^ However, health inequalities persist in Canada despite many public policy proposals and interventions to reduce them.^8–12^ Canadians are concerned about their working, living, and health conditions and the excessive mortality, morbidity, and actual suffering associated with these conditions.

The following statistical data show the extent of preventable health differences among classes and groups in Canada. For example, comparisons of infant mortality rate (IMR) are available. The IMR is one of the better population health indicators because it is specifically sensitive to living and working conditions.^13^ The IMR risk conditions account for the social determinants of health (SDH), such as unemployment, inadequate housing, food insecurity, low maternal education, lack of health care access, and poverty. In Canada, excluding Ontario (no data available), the IMR/1,000 live births for the lowest income quintile was 4.7, while for the highest income quintile, it was 3.2 from 2008 to 2011.^14^ This means that differences in income shape the differences in IMR levels among various social classes. Notably, income is inversely related to IMR: The higher the income, the lower the IMR; the lower the income, the higher the IMR.

Data are also available for other health outcomes and risk conditions associated with housing and food security issues. In Canada, ∼235,000 persons are de-housed or homeless in any given year, of which indigenous persons are overrepresented.^15^ Contrary to some beliefs that people become homeless due to individual choices, laziness, and drug addictions, 78% of the homeless persons in Toronto reported that unemployment, low income, and high housing costs are the primary reasons for their situations.^16^ Unsurprisingly, compared to the general population, they are more likely than other Canadians to experience depression 2×, diabetes 2×, cancer 4×, heart disease 5×, and HIV/AIDS 300 × .^16^ Moreover, the remaining life expectancy at age 25 for homeless women was 5 years shorter than the lowest income quintile of Canadians and nine years shorter than the highest income quintile. For homeless men, remaining life expectancy was 6 and 13 years shorter than Canadians’ lowest and highest income quintiles.^17^ Homelessness is the most extreme example of the broader risk condition of housing insecurity.

Canada's federal government reported that 1.7 million Canadian households were housing insecure, of which 55% were women-led in 2018.^18^ These households did not meet at least one of the following housing standards: *affordability*, that is, housing costs below 30% of the total household income before tax; *suitability*, that is, housing with sufficient rooms for the entire household; and *adequacy*, that is, a well-maintained housing.^18^ As a result, the people living in substandard unaffordable, unsuitable, and inadequate housing are more likely to suffer isolation, stress, injury, asthma, infectious diseases, and cardiovascular-related deaths compared to those who meet the housing standards.^19^ In addition, housing insecurity is highly associated with food insecurity and hunger: inadequate access to nutritious foods due to insufficient income.^20^

In Canada, 1.8 million households were food-insecure in 2017 to 2018. Food insecurity for children was highest in Nunavut at 79%. Other food insecurity levels were as follows: blacks 28.9%, indigenous 28.2%, Arabs and West Asians 20.4%, multiple origins 16.7%, South Asians 15.2%, and East and Southeast Asians 11.3%. Food insecurity for white persons was the lowest at 11.1%.^21^ In Ontario, the mortality rates for the marginally, moderately, and severely food-insecure were 28%, 49%, and 160% higher than for food-secure persons.^22^ This means that generally, food insecurity for low-income people and food security for high-income people partly shapes the mortality differences between them.

The studies above demonstrate that the poor, low-wage working-class, racialized, and women face higher risks of poverty, housing insecurity, homelessness, food insecurity, and adverse health outcomes. Many of the health inequalities they experience are health inequities—preventable differences in health. It is a fact: Although Canada is one of the most advanced capitalist economies globally, class, race/ethnicity, and gender health inequities remain pervasive in this country. This suggests that Canada's state policies failed to address health inequities and their underlying mechanisms. Thus, exposing and challenging the fundamental causes and finding solutions to persistent health inequities are imperative. This task becomes even more critical in light of the highly differentiated impacts of COVID-19 upon those already experiencing health disadvantages.

## Theoretical Framework

The critical political economy theory fundamentally informs this research. The critical political economy approach to health integrates the historical, economic, political, and cultural dimensions of social life into its analysis. It examines the dynamics between class, gender, and race relations; materials and ideas; and social structures and agency shaping health outcomes.^23^ This analytical lens investigates the market, labor, civil society, and state forces influencing policymaking processes shaping the distribution of social determinants of health. It scrutinizes power imbalances among classes, groups, and societies that impact the production and distribution of economic and other resources through the politics of public policymaking by which peoples’ health is shaped.^24,25^ The critical political economy also accounts for sexism and racism structured within and through economic and political relations shaping social and health inequities.^26–32^ It further dissects the role of neoliberalism in creating and maintaining health inequities.^33–36^ This theoretical framework exposes the class interests, ideologies, and power dynamics underlying the state structures that dominate health politics but act little to reduce and end health inequities.^11,37^

The critical political economy theory of health focuses on social relations and the balance of power among social forces of production influencing the distribution of societal resources (e.g., economic, political, cultural, ecological resources) through policymaking processes, shaping individual, family, community, and population health outcomes. The foundation of all human societies is social relations: no social relations, no human societies. Thus, the critical political economy approach to understanding and responding to health and policy change is a helpful analytical lens for capturing the realities of health inequities, their causes, and potential solutions.

## Research Problems

This qualitative research aims to address why and how health inequities, especially class, race/ethnicity, and gender health inequities, persist in Canada and how such differences can be reduced through public policy actions.

## Methods

There is no universal method but rather numerous research methods. Research methods fundamentally involve collecting, analyzing, and interpreting data.^38,39^ The data sources in a qualitative study may include the *document data* (i.e., existing literature and policy documents) and *interview data* or interviews.^38,39^ To answer the research questions, I performed a critical realist review of document data and a thematic analysis of the interview data corpus (briefly explained below due to space limitations).

A developing type of literature review, a ***realist review***, which offers an “explanatory analysis aimed at discerning what works for whom, in what circumstances, in what respects and how,” has been proven helpful in systematically synthesizing complex social and policy interventions concerning health and health care issues (40 p. 21). Specifically, a ***critical realist review*** engages “with realist ontology as well as normative issues that underpin a critical realist approach” that goes beyond what is empirically observable (41 p. 332). This literature review applies social science theory to investigate social practices, including but not limited to health practices, policies, and interventions, accounting for their historical, economic, political, and cultural dimensions and power relations that shape and reshape them.

A critical realist review is a distinct, literature-based methodological approach to critical analysis, bringing in theoretical development or *conceptual innovation* to phenomena under examination (41 original emphasis)—in this case, the critical political economy approach to health inequities. Flexible and open to possibilities and alternatives, it is incongruous with the prescriptive approach to literature review espoused by some uncritical, rigid positivists and empiricists.^41^ There is no single way to perform a critical realist review.

My critical realist review of document data focuses on the political economy of health inequities. This endeavour is important because I expect the interviewees to apply political economy theory in their thinking about health inequities and policy change. Furthermore, critically reviewing classic and contemporary literature on the political economy of health is crucial because literature review connects the study to the broader continuing discourse, fills research gaps, and expands upon previous studies. Finally, a literature review provides the context for establishing the study's importance and a reference for comparing findings.^38,39^ My findings in the critical realist review of literature inform the entire research process, including but not limited to conducting the interviews and analyzing data. I also used findings from existing literature as data sources to reflect on interviewees’ discussions about social and policy change in relation to health. Thus, the literature review and semi-structured interviews inform each other.

I performed 23 semi-structured interviews with Canadian policy academics, activists, and advocates. All are influential and leading experts in their respective fields. My purposive sampling is partly stratified.^38,39^ I used purposive sampling to recruit informants with expert knowledge about social inequities, SDH, and health inequities. Also, this research partly utilized chain sampling.^38,39^ A few informants recommended potential interviewees, and I interviewed three of them.

The recruitment process came in three waves—January, February, and March 2020—with a total acceptance rate of 55% or 23/42 informants.

*Equity, diversity, and inclusion:* While I plan equal representation, the outcome consists of 15 males and eight females, 18 presented as visibly white, and five presented as people of color. I did not ask about the informants’ gender and ethnicity. However, I am aware that at least one female informant is a Métis, and during the interview, an interviewee voluntarily said he is Jewish. This result depended on who accepted, declined, and did not reply to invitation letters. *Homogeneity and heterogeneity*: Each informant can be classified as an academic (6), academic-activist (1), academic-advocate (3), academic-activist-advocate (4), activist (4), activist-advocate (3), or advocate (2). Nine interviewees are health-focused, 10 are non-health-focused, and four are union leaders in the health care industry. Interviewees can also be categorized as a labor leader (4), community organizer and leader (3), sociologist (4), political scientist (4), economist (2), physician (3), epidemiologist (1), housing specialist (1), or public scholar (1).

Before conducting interviews, I secured York University's Ethics Review Board approval certificate STU 2019-135. I did one telephone and eight in-person interviews before the pandemic. Amid the pandemic, five interviews were performed via telephone and nine online. The informants were informed that only audio recording would be done for online interviews. Before the interview, I read the brief background, the purpose of the study, and the informed consent. I sought consent whether or not the participant wished to waive anonymity: 5/23 informants wanted to be anonymous. All informants gave their free and informed consent, some written and some verbal.

After transcribing the audio-recorded interviews, aided by MAXQDA computer software and informed by Braun and Clarke's guidelines, I performed a thematic analysis of the interview data corpus, which can be *inductive* where themes are robustly related to data or *theoretical*, driven by the investigator's analytical interest in the phenomena.^42^ This research attempts to integrate these approaches. Thematic analysis is “a foundational method for qualitative analysis” and can be considered a “method in its own right” (42, p. 78). It is a theoretically flexible approach to qualitative data analysis and could be a realist or constructivist method.^42^ A qualitative approach using thematic analysis based on interviews has been applied in examining preventable health inequalities and has proven useful.^43–45^ Thematic analysis informed by a well-established theory, such as the critical political economy, and supported by empirical data strengthens any research.

## Findings

### A Critical Realist Review of Existing Document Data

Critical political economists explain the causes, barriers, and ways to reduce *social and health inequities based on social relations of production and class relations of power.* For example, Engels showed that compared to the rich and capitalist class, unsafe workplaces, low wages, poverty, inadequate housing, homelessness, and hunger result in high mortality and morbidity for the poor and the working class, in which women, children, older adults, immigrants, and enslaved people are further disadvantaged.^46^ It is a *social murder* because:

“[S]ociety knows how injurious such conditions are to the health and the life of the workers, and yet does nothing to improve these conditions. That it knows the consequences of its deeds; that its act is, therefore, not mere manslaughter, but murder, I shall have proved, when I cite official documents, reports of Parliament and of the Government, in substantiation of my charge” (46, p. 84).

Social murder—social and health injustice arising from inequitable distribution of the SDH—is a logical consequence of capitalism. This is because the bourgeoisie, who owns the means of production, primarily maximize profits and accumulate the nation's wealth in the hands of the monopoly capitalists. As a result, capital accumulation at one end produces a lack of knowledge, oppression, violence, misery, and physical and mental illnesses at the other end, in which the oppressed persons, women, and children are further exploited.^47^ Thus, while Chadwick recommends solving social and health inequities within the legislative halls in the capitalist system,^48^ Marx and Engels propose replacing capitalism with proletarian socialism or communism through social revolution: the core requirement is workers’ unity and class struggle to overthrow capitalism first.^49^

In the twentieth century, after World War II, Keynesian and state-managed capitalism resulted in the so-called Golden Age of Capitalism.^50^ However, those economic and political gains by the working class and subordinated groups eventually eroded as Reagan, Thatcher, and Mulroney promoted deregulated and private capitalism—that is, neoliberal capitalism.^50,51^ International financial institutions such as the International Monetary Fund and World Bank then obliged numerous countries to implement United States-led neoliberal structural adjustment policies. These public policy actions caused the exponential growth of informal workers and slum residents: more than 2 billion informal workers and 1 billion city slum residents.^52–54^ Neoliberal capitalist globalization generated millions of precariat or precariously employed workers, including contractual, temporary, casual, and part-timers.^54^ These workers generally experience higher behavioural, psychosocial, and physiopathological risks than other social classes.^55–58^ Instead of reducing poverty—the single largest determinant of health inequities—neoliberalism exacerbated the working classes’ and slum-dwellers’ toxic working, living, and health conditions, deepening health inequities. It is common knowledge that slum living is unhealthy.

Public policies conforming to capitalism's logic create and sustain health inequities among and within developed and underdeveloped countries. Redistributive public policies, including but not limited to employment security and better working conditions that favor the interests of the working class and subordinated groups in Canada, also occurred. However, regressive policy changes happened because the neoliberal-oriented economic and political elite, gaining hegemonic power, increasingly controlled and directed public policies toward market redistribution mechanisms that more and more satisfied the interests and ideologies of the capitalist class at the local, national, and global levels. Because the capitalist system allows wealth accumulation on the one hand and material deprivation on the other, it results in healthier and longer lives for the few wealthy capitalist countries and the capitalist class and unhealthier and shorter lives for many low-income countries and the working class.^5,33–36,59^ The labor processes under capitalism directly affect workers’ health by producing stress, fatigue, accidents, and toxicity,^60^ such that the heart disease mortality rates for blue-collar workers are two to three times higher than those of professionals and managers, for example.^32^ Regardless of gender and race, most workers suffer health inequities in capitalist societies.

#### The co-constitutive character of class and gender relations further shapes health inequities

For example, globally, women experience higher levels of undernutrition, infectious diseases, cancer, depression, and anxiety and less access to medical care because of poverty. Thus, poverty is gendered.^61,62^ In Canadian patriarchal workplaces, females also suffer more musculoskeletal problems, mental health illnesses, heart diseases, hazardous chemical exposures, cancers, and fatigue than males.^29^ Gendered health inequities remain pervasive in the country's occupational health and health care systems.^28,63^ It persists due to sexist practices and narratives entrenched in a capitalistic political economy and policymaking processes that continuously exploit the historically subordinated females and other sexes; thus, they have more significant morbidities than heterosexual males.^61–65^ For Messing and de Grosbois, addressing social and health inequities requires stronger alliances among researchers and female workers, occupational health scientists and feminists, and working classes and feminist organizations.^29^ Sexism is embedded in capitalism, and it must be addressed accordingly.

#### The co-constitutive formations of capitalism, sexism, Colonialism, and racism further shape health inequities

For example, the colonized indigenous persons experience higher poverty levels, inequitable access to health care systems and services, and mental health problems than non-indigenous persons in Canada.^66^ Furthermore, although labor participation rates for black females and males are higher than the non-racialized, they experience higher unemployment rates and wider pay gaps than the racialized average.^67^ Although Filipino females and males have lower unemployment rates, they experience bigger wage gaps than the average for all racialized workers. These gaps in employment and pay occur because the Canadian labor market is gendered and racialized.^67^ Employment income from racialized and gendered labor market shapes class, race, and gender health inequities in an ensemble, as further evidence suggests below.

Migrant workers and racialized persons experience discrimination. They experience higher levels of precarious working condition, social inequity, and denizenship resulting in health inequities.^31^ The governing authorities failed to address these issues through public policy actions because they succumbed to corporate lobbyists prioritizing private over public interests. In the Canadian health care industry, long before the COVID-19 pandemic, racialized female workers experienced socioeconomic and health inequities due to systemic racism and sexism.^23,26–28,65,68^ Specifically, examining long-term care (LTC) facilities in Ontario, Syed and colleagues conclude: “Our study shows how work hierarchies; rigid divisions of labour, and task orientation within LTC are highly complex phenomenon that can intersect with psychosocial factors. Employee job stress, high job demands, and time pressures… also seem to be linked to the experiences of care work hierarchies and of task-oriented work between and among various worker groups. Care work is gendered and racialized” (69, p. 14)

Another way to understand the critical political economy approach to health and policy change is through welfare state systems analysis that brings to light the political and public policy dimensions of health inequities. For example, it has been found that the mean IMR/1,000 live births were consecutively lowest for the social democratic than for Christian democratic, former fascist dictatorships, and liberal Anglo–Saxon welfare states such as Canada from 1960 to 1996.^70^ This is because, in welfare states governed by social democratic parties supported by strong labor movements, population health indicators were better due to robust redistributive public policies. In contrast, redistributive public policies were weaker in countries where the capitalist power and influence are more substantial than the combined power and influence of the social democratic parties and labor movements.^70^ Specifically, the Christian democratic welfare states such as the Netherlands heavily depended on the family for delivering social services and health care to children, seniors, and persons with disabilities. This extra burden of care work resulted in women's labor participation at only 46% compared with the liberal at 52.8% and social democratic at 65.2%. The former fascist countries have the lowest employment rate, at 26%.^70^ Consequently, in Christian democratic countries, class inequities have been exacerbated by gender inequities, furthering class and gender health inequities. In Spain, females experienced two times as many mental stress-related health conditions as males.^70^

The governing authorities and political parties representing the interests and ideologies of a particular class, through public policy actions and inactions, profoundly shape health outcomes. For instance, Navarro and colleagues show that labor and welfare policies reducing social inequities generally improved the IMR in Organisation for Economic Co-operation and Development countries from 1950 to 1998.^71^ The historical economic and political tradition of social democratic parties with egalitarian ideologies implemented those healthy public policies.^72^ Other scholars demonstrate that the social democratic distribution of economic and other resources improves population health and reduces health inequities,^73^ providing further evidence that consolidated working-class power and socialist party political representation are core requirements in ending health inequities through equitable distribution of the social determinants of health.

The following discussions examine corporate power in relation to health inequities. The power and influence of big business shape public policy, resulting in the inequitable distribution of SDH, including employment, housing, early child development, and health care.^25,73^ In Canada, the corporate-driven neoliberal policies worsen social and material deprivation, resulting in psychosocial stress, unhealthy coping behaviours, and health inequities, in which the poor, low-income workers, females, and racialized persons are further disadvantaged.^12,25,73^ More concretely, in Canada's labor market, the power imbalances among classes and social forces resulted in rapidly increasing profit shares for the capitalists and incremental wage increases for the workers from 1990 to 2010.^74^ This is because, during this neoliberal period, concessionary labor wages and benefits and de-unionization escalated. Moreover, temporary, part-time, and contractual labor became standard employment practices. Finally, the corporate downsizing strategy, which cuts production costs to maximize profits, plus the closures of many small and medium-sized enterprises, resulted in massive joblessness, housing insecurity, and a plethora of social inequities,^74–78^ shaping unequal health outcomes.^73^ In Canada's housing industry, neoliberalism intensifies housing insecurity and homelessness through public policies that adhere to the corporate-led, market-dependent, profit-driven housing provision.^8,79–82^ These neoliberal housing policies resulted in the following. First, the ongoing rise in housing prices far exceeded household incomes. Second, the demolition and conversion of low-rental housing units into luxury condominiums depleted affordable housing stock. Third, high rental costs negatively affected millions of Canadians. Finally, the termination of public and social housing long-term subsidies exacerbated housing insecurity, homelessness, and health inequities,^8,83,84^ severely impacting the working class, females, and racialized persons.^85^

Indeed, Canada's state policies prioritized the profit interest of the business elite over fundamental human needs for a decent living. The managers and owners of the big corporations succeeded in pressuring governing authorities to implement a neoliberal approach to macroeconomic policies favoring the elite 1%.^86^ Consequently, the total wealth of 12 million lowest earners now only equals the wealth of 87 most affluent families in the country.^87^ Canadian life expectancies also widened between the lowest and the highest quintile income earners by an additional one year for males and about 2 years for females between 1996 and 2016.^88^ Capitalism's hallmark is abject poverty and early deaths on one extreme and affluence and longer lives on the other extreme. In the capitalist economic system, women and racialized workers are significantly more exploited than men and non-racialized workers, deepening health inequities. Sexism and racism are engrained in capitalism—these social systems co-constitute each other to a greater degree. Gender and racial capitalism primarily shape class, gender, and racial health inequities.

***What is to be done?*** As discussed earlier, while Chadwick suggests policy reforms within the existing capitalist system,^48^ Marx and Engels recommend a revolutionary class struggle to smash capitalism and replace it with communism or proletarian socialism.^49^ In the contemporary era, some emphasize class, gender, and race relations underlying the economic, political, and cultural structures shaping the SDH and health inequities and propose addressing these issues in an integrated manner.^26,28,30,68^ Exposing, reducing, and ending class, race, and gender health inequities necessitate solidifying labor, social, and political movements to counter the capitalist class and compel the capitalist state to implement public policies that equitably distribute the SDH.^26,71–73^

Wright explains that in the twenty-first century, anticapitalism struggles against social and health inequities may be informed by interacting but distinct strategic logics of smashing, dismantling, taming, resisting, and escaping capitalism.^89^ The capitalist political-economic system underlies public policies that result in the inequitable distribution of the SDH, resulting in health inequities. The capitalist class and the capitalist state predominantly create and maintain health inequities. Socialism is a plausible alternative system to reducing class, gender, and racial health inequities.

### Thematic Analysis of Interview Data Corpus

For the most part, my critical realist review of existing document data supports the results of the thematic analysis of the interview data corpus and vice versa. The thematic analysis of the 23 semi-structured interviews with influential and leading Canadian policy academics, activists, and advocates demonstrate that health inequities in Canada are primarily caused by the capitalist economic system; the co-constitutives of capitalism, namely colonialism, racism, and sexism; and maldistributive public policies. Canada's health inequities reduction requires pushing for redistributive public policies; uniting and strengthening labor unions, civil society groups, and social movements; and engaging in electoral politics. Reducing and ending health inequities in general and class, race/ethnicity, and gender health inequities, in particular, may involve struggling within and against capitalism and struggling for socialism.

### Causes of Health Inequities

#### The capitalist economic system

Although interviewees came from various backgrounds, 10/23 of the informants spontaneously identified capitalism as the fundamental cause of social and health inequities. However, as the interview progressed, 18/23 informants explicitly mentioned capital, capitalism, capitalist, neoliberal, neoliberalism, or neoliberalization as the primary sources of health inequities. Only 5/23 informants did not explicitly use those terms. However, they also speak about the dominant role of the private market system and big business in health politics and public policymaking.

Some interviewees focus on the links between capitalism, employment, and health. For example, Sam Gindin, a political activist, former Research Director of the Canadian Auto Workers for 27 years, and Packer Visitor in Social Justice at York University for 10 years, explained that capitalism primarily shapes low wages, unsafe working conditions, and adverse health outcomes. Gindin emphasized that the “class and the relative balance of class forces” in which the “power of capital and the power of the state” defeated the working class mainly drives social and health inequities:

Capitalism. That's the story… This is what it means to live in a class society. It's unequal for workers, which means not just lower wages, but the pace of work is constantly tightened, which puts pressure on people. It means they live in permanent insecurity, which has all kinds of health and mental health issues… So, it's the basic workings of our capitalist society of one-half of the equation. The second half of the equation is that we haven’t been able to build the kind of social forces that have effectively offset that. I think an obvious answer, it's about class and the relative balance of class forces. And the fact that workers aren’t a class, they’re fragmented individuals, and as fragmented individuals, they don’t have the power to take on the capitalist system, inequalities. And the working class over the last 30, 40 years now has been defeated. So, it's no surprise that we find these results (Gindin: Activist).

William Carroll, a Professor of Sociology at the University of Victoria since 1991, also unequivocally stated that capitalism, or global neoliberal capitalism in its current form, is the fundamental cause of persistent social and health inequities. He explained: “I would say that all of those inequities are primarily caused by class relations and, in particular, the social structure of what we might call advanced capitalism. And it's capitalism that's globalized now and neoliberal, and it's a way of life that basically involves this structural divide between those who own capital and control capital and those who work for wages and salaries. And it's a system that is driven by the class that owns and controls capital. And its interests are primarily in maximizing profit and accumulating capital” (Carroll: Academic).

#### The co-constitutives of capitalism: Colonialism, racism, and sexism

Spontaneously, 6/23 interviewees said the cause of social and health inequities are co-constitutive class, nation, race/ethnicity, and gender relations. 1/23 informants explicitly mentioned *intersectionality*. No informant stated right away that sexism or patriarchy alone causes health inequities. However, as the interview progressed, 23/23 informants—explicitly or implicitly—linked health inequities to the co-constitutive character of capitalism, colonialism, racism, and sexism. For example, Pat Armstrong, a Professor of Sociology at York University and Fellow of the Royal Society of Canada, explained the gendered, (neo) colonial, and racialized labor market system shaping health outcomes:

One of the most important things is capitalism—an economy based on the search for profit… And that, in turn, leads to the kinds of issues and employment you were talking about and inequalities… In terms of the gender issue, I think that it's certainly not exclusive to capitalist societies. But that the search for profit has taken advantage of gender differences in gender assumptions and reinforced them in ways that perpetuate inequality. And the same can be said of race, especially building economies around racial differences that emphasize those inequalities… [W]e see racism perpetuated by the use of labor from other countries to import and to bring in, as John Porter said, a long time ago at the bottom and to fill the jobs at lower pay and with less power (Armstrong: Academic-Activist-Advocate).

Anonymous N7 further explained the co-constitutive class, nation, race, and gender relations shaping health inequities through and within precarious work: “[T]he capitalist system is producing all these precarious jobs. The fact that it's a system that is based on maximizing profits, and profits are maximized in one sense, by cutting labor costs… Like you can substitute labor with technology, or you can take your company to Global South country where labor is cheaper… or you can hire labor here in Canada, which is ideologically labelled as being inferior labor because of racialization and sexism and other ideologies that define certain types of labor as inferior labor. And therefore, the rationale is created to pay them less or keep them in a more insecure situation” (Anonymous N7: Academic).

Anonymous N7 illustrated the relationships between class, race, migration, and education: “[M]igrant workers are kind of kept in a captive situation because they are here only to do labor. And after they finish the labor, they will be sent back to their country. And they’re usually coming. And also, people who are from poor backgrounds, like people who have experienced poverty, don’t have a lot of options. And also, if, for instance, a person has an education that has not been recognized or does not have an education background, does not speak the official language, their options are less like their alternatives are less. So, they have been produced as precarious labor through racialization, through gender ideologies, through their migration status, through exclusionary processes from educational institutions and so on.”

#### Maldistributive public policies

5/23 of the informants spontaneously responded that maldistributive public policies are the causes of social and health inequities. However, as the interview progressed, 23/23 interviewees recognized the failures and limitations of public policies within a capitalist economic system. For example, Sheila Block, a Senior Economist at the Canadian Center for Policy Alternatives–Ontario, who works primarily on the labor market and public finance, explained that social and health inequities result from inadequate government policies around pre-distribution (e.g., minimum wage), redistribution (e.g., taxation), and provision of public services or social wage:

One of those fronts is what's called pre-distribution. So, it's how is the private-sector economy regulated? Part of that regulation has to do with minimum standards, like minimum wage legislation… And how do those policies contribute to inequality in the area of pre-distribution? The second area is around what's often called redistribution. And what that is, is the impact of tax policies. How much tax policies reduce inequality? And while our tax system does reduce inequality, it doesn’t do it sufficiently. And then the other piece of that is around the provision of public services, which is often called the social wage. Social wage is more important to low-income individuals than to high-income individuals. So, I think those kinds of three buckets of public policy make an important contribution to the lack of progress on reducing income inequality (Block: Researcher-Advocate).

Cathy Crowe, a member of the Order of Canada, founding member of the Shelter and Housing Justice Network, and Distinguished Visiting Practitioner at Ryerson University, pointed to the termination of public and social housing: “[T]he policies of neoliberalism… two federal governments, over ‘93, ‘94, Conservative and Liberal, cancelled our national housing program. And when that happened, we lost the production of 20,000 new units of social housing a year across the country.” Block corroborated Crowe's insight about some redistributive public policies that are susceptible to regressive policy reversals: “I think some of the reasons for these failures have to do with the power of capital… An example of that was Bill 148, the Amendments to the Employment Standards Act and the Labour Relations Act… And because this policy came just before an election year, there were many reversals… a very sizable increase in the minimum wage from, I believe, $12 and change to $14. However, we didn’t have the next step to $15, which was supposed to occur. And many of their changes, including paid sick days, which were for emergency leave days, which of course, the lack of that is having terrible impacts. Reversal was there as well. So, I think the power of vested interests has a big impact.”

### What Should Be Done: Toward Emancipatory Social Change and Health Justice

#### Pushing for redistributive public policies

The collective insights of the informants are pushing for at least 39 public policy recommendations to reduce social and health inequities in Canada, most of them around the equitable distribution of the social determinants of health. On top of the proposals, 12/23 to 14/23 informants recommend improving taxation, employment and working conditions, social support, income support, and health care. In the middle, 6/23 to 11/23 informants suggest addressing housing and homelessness, pharmacare, education, childcare, poverty, unionization, and climate crisis. Finally, no more than 5/23 informants propose electoral reform, disability benefits, public transit, food security, anti-discrimination, senior and retirement benefit, migration and refugee support, permanent status, participatory democracy, dental care, rent control, recreation, government coordination, de-commodification, nationalizing finance, Green New Deal, Just Transition, degrowth, healthy urban living, small business support, anti-scab, undocumented workers support, race-based data collection, de-stigmatization, clean water, internet access, and rehabilitation.

Because most of the above policy proposals have long ago been forwarded elsewhere and in Canada, and due to space limitations, I only present a few illustrative quotes. For example, Sharleen Stewart, President of SEIU Healthcare, proposes a “full-time job that provides a decent living.” Political scientist Greg Albo suggests: “particularly dealing with unemployment.” Sociologist Anonymous N7 recommends policy changes to improve the working and living conditions of the temporary migrant workers: “I think for migrant workers, we need to have permanent residency. If they’re working here for years, then we need to give them legal status. Otherwise, we need to tighten up the minimum labor standards like minimum wage, overtime hour… They should be expanded to cover contract workers” (Anonymous N7: Academic).

Cathy Crowe suggests sufficiently funding national, provincial, and municipal social and public housing programs for extended periods to address housing insecurity and homelessness and the adverse health outcomes associated with them: “Securing adequate shelter and funding for shelters is critical. Berlin… created a rent freeze… We need… vacancy tax and more inclusionary zoning. But those are just still Band-Aids compared to having targets to build X number of thousand social housing units per year… Increasing social assistance rates and minimum wage rates as well so that people can actually afford their various social determinants of health, including food.”

#### Uniting and strengthening labor, civil society, and social movements

23/23 informants—explicitly or implicitly—suggest uniting and strengthening labor, civil society, and social movements to push for redistributive public policies addressing the inequitable distribution of the SDH to reduce health inequities. For example, John Clarke, a retired organizer of Ontario Coalition Against Poverty, Packer Visitor in Social Justice at York University and a long-time activist, argues that changing the social system that favors the interests of the elite instead of the masses requires uniting and mobilizing peoples' movements. A united movement of movements is necessary to challenge the dominance of the capitalist class and Conservative and Liberal parties in institutional public policymaking processes. Beyond that, one may see a spiralling social decay before the governing authorities address pressing social and health problems: “I believe that primarily movements and social mobilization and communities that are active and struggling and challenging are the only way to put any limits on what they can achieve. Outside of that, you can only hope that things reached such a level of social dislocation, such a level of public health crisis, and they’re forced to take some measures” (Clarke: Activist).

Cathy Crowe states that a massive national movement composed of labor unions, non-profit organizations, and faith communities, among others, is again necessary to fight for public policies that can reduce social and health inequities: “It was a very vibrant, popular movement supported by everybody, supported by unions, supported by foundations, supported by faith groups. And ultimately, that had a huge win that a lot of people don’t know about… And it was a national movement that led to the first national housing program. And my argument was, that's what we need again now to deal with the problem” (Crowe: Activist).

It is essential to report that while some of the informants implicitly suggest uniting and strengthening peoples’ movements should be forged against capitalism being the fundamental cause and barrier to reducing and ending health inequities, others openly express that part of the solution to class, race/ethnicity, and gender health inequities is going beyond capitalism. For example, William Carroll states: “I think class, race and gender and ethnicity are all sort of bundled together, but I do tend to see the structure of capitalism as the primary driving force. And it actually historically has incorporated relationships of race, ethnicity, and gender in certain ways. It reproduces those inequalities. Well, you’re probably going to ask me about what the solution is. I think, obviously, part of the solution is getting past capitalism. But, I think one also has to at the same time address these other inequities” (Carroll: Academic).

Trevor Hancock, a retired Professor and Senior Scholar of Public Health and Social Policy at the University of Victoria, a former consultant at the Ministry of Health in British Columbia, and Green Party first leader in both Canada and Ontario, explained:

[W]e have to recognize that what we’re seeing is the end of an age of capitalism and particularly neoliberal capitalism, capitalism in general. Because the inevitable consequence of this form of capitalism is, on the one hand, oligarchy, which I talked about earlier. And, on the other hand, the destruction of the Earth's resources. It's the logical consequence of our form of capitalism, and it's not sustainable. And if we don’t stop it, then society will collapse sometime in the next 50 to 100 years… I think where we focus is not so much on criticizing the current system as building the better one… And the better can’t be a kind of socialist paradise version of the current capitalist system, because the current capitalist system, it doesn’t really matter whether it's state capitalism like Russia, or free enterprise capitalism like the [United States], but what used to be state capitalism, they both treat the Earth as something to be used up. They actually both create their inequalities in their own way. We’re going to have to transition to something that is radically different fairly quickly (Hancock: Academic-Advocate).

#### Engaging in electoral politics

4/23 informants explicitly propose electoral reform to increase political representation in institutional public policymaking processes. They believe in participating in electoral politics to elect progressive and socialist-oriented policymakers with the political agenda to enact socioeconomic policies that can address the inequitable distribution of the social determinants and health inequities. For example, Toba Bryant, a Public Scholar and Professor specializing in social determinants of health and health policy, suggests: “Electoral reform would help. We need bolder political parties. Jagmeet Singh ran a reasonable campaign in the last federal election, and they need to do more of that. They need to be a little more upfront and be more explicitly focused on promoting redistribution in our social and health policies and economic policies. But making clear, I think people need to be informed about the issues, and they need to be encouraged to vote and participate” (Bryant: Academic).

While some support the New Democratic Party (NDP), other informants are ambivalent. For example, Carroll states: “Political parties are important. I don’t know if the NDP will ever be the party that could really enact these kinds of changes. I’m doubtful, but it's the party on the left that we have now. So, one possibility is to try to push the NDP in a stronger direction.” Epidemiologist Anonymous N14 suggests: “It's the combination of the window of opportunity with broad political coalitions that have a common interest in reducing health inequities. And not through traditional parties. Maybe pressuring them just from the street. There is no other means.” Sam Gindin points to the challenges faced by the left-leaning forces and the opportunities offered by the Corbyn and Sanders phenomena in the field of electoral politics: “People are frustrated with their lives… So, the question is, what happens to that frustration? The left hasn’t been able to take advantage of that yet. You have signs, Sanders, Corbyn, but generally, the left has failed. Social democracy has failed to speak to those frustrations because they tried to take a middle road. And that failed… The failure of the left is very dangerous. It's opened it for the right… And the challenge is for the left to be able to take on this challenge. You know, as Corbyn tried to do, and Sanders tried to do. We shouldn’t get discouraged. This is just the beginning” (Sam Gindin: Activist).

Greg Albo observes the absence of political parties waving the flags of communism, socialism, or social democracy that advances alternative politics toward a new society with a socialist political-economic system where redistributive public policies to reduce social and health inequities are more plausible: “[W]e’re dealing with this really difficult historical period and the history of capitalism and the history of the left where we don’t have ascended political parties carrying the banners of an alternative society. And an alternative society going into the names of socialism or communism and not even one that carries the banner of historical, social democracy. And that is, we’re going to tax the rich enough so that we have free public provisioning of health care, free public provisioning of transport, free public education, and so forth. And we’re going to make those better every year, and we’re going to do it in a way that's reducing income inequality. We don’t even have that anymore” (Albo: Academic-Activist-Advocate).

## Discussions

### Causes of Health Inequities

#### The capitalist economic system

The main finding from the thematic analysis of the interview data corpus, that capitalism is the fundamental cause of health inequities, is not new. However, most striking is the informants’ common insight—expressed more explicitly than implicitly—that capitalism or neoliberal capitalism is the primary mechanism driving health inequities in Canada. No informant stated that genes, behaviors, and lifestyles basically produce health inequities. This result is significant because this research is probably the first to gather diverse, influential Canadian policy academics’, activists’, and advocates’ insights about social and health inequities.

As presented in the critical realist review, the working class has experienced higher morbidity and mortality rates for centuries than the capitalist class because of the capitalist economic system that inherently exploits the workers for profit maximization and wealth accumulation, favoring the capitalists.^46,47^ Since the 1970s, neoliberal capitalism has produced billions of informal workers worldwide.^53,54^ These precariously employed workers experience higher physical and psychosocial health risks than other classes and groups. Precarious employment primarily impacts manual laborers, young workers, workers with low education, immigrants, and women.^55–58^ Precarious work deepens health inequities.

Worse, amid COVID-19, while the Canadian billionaires’ aggregate wealth soared by $78 billion, about 5.5 million workers lost their jobs or reduced their working hours.^90^ Socioeconomic conditions such as employment, income, and living conditions determine health outcomes; thus, it is unsurprising that the COVID-19 mortality rates for the lowest income quintile earners are two times higher than those of the highest income quintile earners.^91^ Canadians with the lowest income, those living in apartments in urban areas, and neighborhoods with more visible minorities recorded 14 to 30/100,000 more deaths than their counterparts.^92^ Under capitalism, with or without a pandemic, the poor and low-income workers experience more significant economic and health risks than the wealthy and capitalist class. Capital accumulation and concentration in the few hands create penury for many.

#### The co-constitutives of capitalism: colonialism, racism, and sexism

The collective insights of the informants related to this second finding bring to mind three stark realities. First, the inextricably interrelated capitalist-racist-sexist system primarily shapes the ensemble of class-race-gender health inequities. For example, in the health sector, the capitalist state policy interventions maintain the class structure where physicians are mostly upper-middle-class, white males; nurses are primarily lower-middle- and working-class females; and auxiliary workers are mainly females from working-class backgrounds.^93^ As such, state policy actors perpetuate the mechanistic-individualistic ideology of medicine that complements rather than opposes the ideology of capitalism. Furthermore, the political and economic elite maintain the capitalist mode of production that primarily drives workers’ alienation, adversely impacting the latter's well-being.^93,94^ Nonetheless, class struggles to change these exploitative-oppressive conditions mark the history of the working classes and subordinated groups worldwide.^5,35,60,94^

The typical work arrangement in Western health care systems dominated by capital-oriented policymakers and upper-class, non-racialized, heterosexual, male physicians points to the co-constitutive character of the class, race, and gender dynamics. Not surprisingly, amid the pandemic, the nurses and auxiliary workers in the health sector suffered more than other classes and groups. There is a need to address class, race, and gender relations in an integrated way to effectively address social and health inequities.

Second, the intricately interconnected capital-colonial-racial system mainly shapes class and racial health inequities. For instance, public policies, laws, economic arrangements, and institutional practices perpetuating indigenous ancestral land grabs, socioeconomic inequities, environmental racism, racial violence, prolonged exposure to psychosocial stress resulting in adverse physiopathological outcomes, and culturally insensitive health care systems and services create and sustain health inequities.^95,96^ As a result, the life expectancy difference between Nunavut and Canada's average is ∼10 years, for example.^97^ Part of the reason is that the indigenous persons suffer from the intergenerational, adverse health effects of the capitalist, state-sanctioned, residential school system.^98^ Moreover, they are 10 times more likely to use shelters for the homeless than non-indigenous people.^99^ Despite extensive evidence that homelessness kills, this problem remains widespread in Canada.

Furthermore, Canada's food insecurity level for blacks is three times higher than for whites,^100^ partly because people of color generally earn 30% less income, and their poverty rate is twice that of the white ethnicity.^101^ Racism is entrenched in the Canadian labor market. Racism and colonialism, entangled with capitalism's inherent tendency to cross continents in search of cheap labor and resources, market expansion, and capital accumulation, underlie the disadvantageous material and social conditions of the (neo) colonized and racialized societies and groups. Racial capitalism fundamentally produces and maintains class and racial health inequities.

Finally, the internally intertwined capitalist-sexist system predominantly shapes class and gender health inequities. The pre-existing class and gender inequities and the uneven economic and health impacts of COVID-19 experienced by low-income women can be linked to global capitalist and patriarchal practices concentrating the wealth and power in the hands of extremely few elite males.^26^ Evidence shows that the aggregate wealth of the 10 wealthiest men is greater than that of 40% poorest population globally, at the bottom of which are impoverished women and girls.^102^ This gendered global wealth inequity results from a patriarchal economic system that undervalues female wage labor in the production of social life, such as caring and domestic labor. Neoliberal policies failed to address the gender pay gap such that from 100 years, it will now take 136 years to close it.^102^ In Canada, the policy shift from Keynesianism to neoliberalism weakened social support, aggravating violence against women.^103^ Gender capitalism fundamentally creates and sustains class and gender health inequities.

The co-constitutive spheres of (neoliberal) capitalism, (neo) colonialism, racism, and sexism determine class, race/ethnicity, and gender health inequities. These analytically distinct but inherently interconnected interacting social systems adversely impact the colonized, racialized, and subordinated sexes’ working, living, and health conditions. Gender and racial capitalism determines the inequitable distribution of SDH, resulting in unjust health inequalities.

#### Maldistributive public policies

The collective insights of the informants affirm that public policies, especially under neoliberal governments, failed to distribute the social determinants of health equitably to reduce health inequities. This finding is not new. As mentioned earlier, Canada's housing insecurity affected 1.7 million households, 55% of which were women-led in 2018.^104^ Moreover, food insecurity impacted 1.8 million households, including 1.2 million children from 2017 to 2018.^21^ Canada's child poverty rate was 11.8%, compared to Norway's 8.1% and Finland's 3.5% in 2018.^105^ These statistics show that Canadian state policies are failing and significantly lagging behind the social democratic countries.

Furthermore, it is essential to emphasize that existing redistributive public policies are subject to reversals. For example, Ontario's Conservative government terminated the basic annual income pilot project, ignoring that the MINCOME experiment in Manitoba has improved the social and health conditions of those living in poverty.^106^ It dissolved the Roundtable on the Violence Against Women advising the province on women's violence-related issues.^107^ The Conservative government also implemented numerous health care funding cuts documented by the Ontario Health Coalition,^108^ eroding Canada's supposedly universal health care system. The collapse of the Canadian health care system—for example, the severe staff shortages resulting in increased workloads, closures of emergency services, cancellations of surgical procedures, and high levels of COVID-19 infection rates among health care workers, which endangers people's health—can be linked to governing authorities’ affinity to neoliberalism despite outcomes of widening inequities.

### What Should Be Done: Toward Emancipatory Social Change and Health Justice

#### Pushing for redistributive public policies

The shared insights of the informants related to employment, migrant status, and housing policy change to reduce health inequities help recall that amidst the COVID-19 epidemic in Canada, 296,000 persons lost their jobs, and 247,000 persons lost half of their working hours from February 2020 to March 2021,^109^ further disadvantaging the mostly racialized 1.7 million migrant workers, students, and refugees, 500,000 of whom are undocumented.^110^ Moreover, Canada's housing-insecure households struggled with soaring housing ownership and rental costs, the homeless were violently displaced from encampments, and food-insecure persons drove in flocks to the food banks. As a result, these people face further economic and health risks. For example, although Wilkinson ignores class relations and neoliberal policies shaping social and health inequities,^26,111^ Wilkinson demonstrates that unemployment and employment income are causative factors of relative poverty and mortality differences among social classes.^112^ As mentioned earlier, the working class, especially the precariat and the unemployed, face higher morbidity risks than other social classes. Improving employment and working conditions and increasing income to address housing and food insecurity can reduce health inequities.

There is no shortage of evidence-based research and policy proposals addressing the inequitable distribution of SDH and health inequities, including class, race, and gender health inequities (e.g., see 4–12, 23–36). However, as discussed earlier, redistributive public policies that can help reduce, if not end, health inequities are severely constrained in a capitalist system. Thus, there is a need to go beyond capitalism—this exploitative-oppressive social system inherently prioritizes profit and wealth accumulation over everything.

#### Uniting and strengthening labor, civil society, and social movements

The informants unanimously acknowledge that the labor unions, civil society groups, and social movements are fragmented and weak at the present moment to prevail over the capitalist class and neoliberal governing authorities dominating health politics and public policy. Logically, they suggest uniting and strengthening these peoples’ movements to effect social and policy change. The informants affirm that collective struggles of peoples’ movements can bring about redistributive public policies necessary to reduce health inequities.

Aside from the lived experiences shared by the informants, among other collective forms of struggles to improve people's working, living, and health conditions, one may learn lessons from the historic Ontario's Days of Action, Maple Spring, and Alberta's Nurses Strike. Moreover, successful stories of people's power over corporate and state power are well-documented worldwide. In the health field, one should recall how united, organized, and informed advocacy and activism successfully built health-centered social movements, such as the Women's Health Movement, Breast Cancer Activism, AIDS Coalition to Unleash Power, and Needle Exchange Program. These health-based social movements *from below* powerfully influenced social and health policy legislations to reduce health inequities between classes and groups across and within countries.^113^

Labor, civil society, and social movements can improve population health through redistributive public policy advocacy and activism. There are thousands of labor unions, civil society groups, and social movements comprising millions of members, vastly outnumbering the neoliberal state and corporate policy actors. However, because these peoples’ movements are weak and fragmented, they cannot overcome the power of the more organized economic and political elites. This fundamental weakness of the peoples’ movements should be addressed urgently amid multiple existential crises: deep inequities, ecological destruction, recurring epidemics and pandemics, right-wing authoritarian populism, and militaristic imperialism that severely impact the historically exploited-oppressed classes, groups, and nations.

#### Engaging in electoral politics

As presented earlier, some informants proposed participating in electoral politics. The results demonstrate that a socialist political party as an alternative to traditional parties may be required to push for redistributive public policy within the capitalist system or beyond capitalism to establish socialism to realize social justice and health equity. Thus, I further engage Wright's propositions concerning anti-capitalism struggles in the twenty-first century.^89^

First, engaging in health politics and the political economy of health inequities can help *smash capitalism* by capturing state power through elections and the right to suffrage. Second, engaging in health politics and public policymaking can *dismantle capitalism*, a passage to democratic socialism through state-led reforms reducing the harms of unbridled capitalism. Smashing and dismantling capitalism envision replacing capitalism with socialism to reduce health inequities.

Third, engaging in electoral politics and healthy public policymaking processes can *tame capitalism* through social democratic parties and non-revolutionary socialist parties. Social democracy tames and resists capitalism, although it merely counteracts the harmful effects of capitalism rather than dismantling capitalism itself. Dismantling and taming capitalism necessitate collective struggles by organizations, expressly political parties, to capture and exercise state power to reduce health inequities.

Fourth, *resisting capitalism*, the primary form of struggle of most labor unions, civil society groups, and social movements, which operates outside the state without attempting to capture state power, may help reduce class, race/ethnicity, and gender health inequities by pushing for redistributive public policies. Fifth, *escaping capitalism*, which disengages from politics, may protect one's well-being. While resisting and taming capitalism counterbalance the adverse health impacts of capitalism, escaping, dismantling, and smashing capitalism go beyond the structures of capitalism to reduce health inequities.

Lastly, reducing health inequities by *eroding capitalism* combines state-led efforts to dismantle and tame capitalism with peoples’ movements’ efforts to escape and resist capitalism. Outside Canada, this strategic logic is exemplified by the Syriza, Podemos, Sanders, and Corbyn phenomena, as also mentioned by some informants. More recent political developments show that campaigning for more democratic socialist policies and government platforms can win the broader support of the working classes and other historically subordinated groups. In Latin America, from the working classes and marginalized groups, Brazil's Lula da Silva, Bolivia's Evo Morales, Peru's Pedro Castillo, and Chile's Gabriel Boric. In the United States, The Squad.

Engaging in electoral politics is crucial because, in parliamentary democracy, registered voters choose their representatives to legislate laws concerning the distribution of economic, political, cultural, and ecological resources in which peoples’ health is shaped. At the present historical conjuncture, considering the relative balance of power between class and social forces tilted in favor of the capitalist-oriented forces in and outside the state, in my view, eroding capitalism is a promising alternative strategy to reducing health inequities in general and class, race/ethnicity, and gender health inequities in particular. However, one must not ignore the reality that genuine emancipatory social and policy change emanates *from below*.

## Some Research, Policy, and Practice Implications

Researchers of inequities in health and health care should integrate into their research, policy, and practice the often marginalized, if not ignored, critical political economy approach to understanding and responding to persistent social and health inequities in Canada and elsewhere. By going beyond the biomedical model of health that emphasizes genes, lifestyles, and behaviors as the primary sources of unequal health outcomes, researchers can better expose, critique, and alter the dominant power structures that create and perpetuate health inequities and can thereby favor the less powerful and subordinated classes and groups across and within countries.

Academics in general and policy-oriented academics, in particular, should realize that the better way to tackle persistent inequities in health and health care is by collaborating with activists and advocates who are more proximal, if not directly grounded with, the people who experience social and health inequities. Social justice activists and advocates should consider the importance of integrating the pressing issues of inequities in health and health care in their collective struggles. By working together, they can craft innovative policy changes and practical actions around the SDH that can better improve individual, family, community, and population health outcomes.

Politicians, public policymakers, and government authorities should thoughtfully examine the persistence of inequities in health and health care and reconsider their positions about the market-oriented, profit-driven economic system. In capitalism, redistributive public policies aimed to improve the lives of the exploited and oppressed classes and groups are very limited and incessantly subjected to regressive policy reversals. The capitalist system kills people on a massive scale. There is a need to democratize governance to realize more socialist-oriented research, policies, and practices to effectively address the widening social and health inequities that threaten Canadians and human societies.

Transformative social change necessary to reduce health inequities will not be delivered *from above* by the economic and political elites who adhere to the principles of capitalism. On the contrary, world history shows that emancipatory social change often comes from the unified struggles of the peoples’ movements *from below*. Thus, progressive and socialist-oriented labor unions, civil society groups, social movements, and political parties should solidify and strengthen their ranks to countervail and overcome the power of the capitalist classes and the capitalist states destroying the ecology, communities, livelihoods, and people's lives.

## Conclusions

Health inequities are existential societal problems partly shaped by public policies enacted and implemented by the governing authorities. The phenomena persist despite vast public policy proposals and interventions to address such inequities among and within countries. This research aimed to answer how and why health inequities, especially class, race/ethnicity, and gender health inequities, persist in Canada and how such differences can be reduced. Theoretical-empirical evidence demonstrates that the causes of health inequities are primarily structural, institutional, and ideological, occurring at the macro-societal rather than micro-individual level. The critical political economy approach to health and policy change is useful in examining health inequities, as evidenced by the distinct but interrelated findings in this research.

The reasons why health inequities, especially class, race/ethnicity, and gender health inequities, persist in Canada are the capitalist economic system; the co-constitutives of capitalism, namely colonialism, racism, and sexism; and maldistributive public policies. The answers to how health inequities can be reduced in Canada are pushing for redistributive public policies; uniting and strengthening labor, civil society, and social movements; and engaging in electoral politics. Reducing health inequities in general and class, race/ethnicity, and gender health inequities, in particular, may involve struggling within and against the capitalist system and struggling for socialism.

There has been little research on the understanding that public policy academics, activists, and advocates hold on the importance of the inequitable distribution of social determinants of health that create health inequities and the means of addressing them. This research identified the fundamental causes and ways to reduce health inequities by inquiring and analyzing their insights. This study contributes to current scholarly and public debates on reducing health inequities. It may provoke social actions toward transformative social change to achieve health justice.
